# A Promising Ash Supplementation Strategy in the Cultivation of *Spirodela polyrrhiza* Plants

**DOI:** 10.3390/cells12020289

**Published:** 2023-01-11

**Authors:** Zdzisława Romanowska-Duda, Krzysztof Piotrowski, Dariusz Stępiński, Katarzyna Popłońska

**Affiliations:** 1 Department of Plant Ecophysiology, Faculty of Biology and Environmental Protection, University of Lodz, Banacha 12/16, 92-237 Lodz, Poland; 2 Department of Cytophysiology, Faculty of Biology and Environmental Protection, University of Lodz, Pomorska 141/143, 90-236 Lodz, Poland

**Keywords:** root meristem cells, nucleoli, sorghum ash, *Spirodela polyrrhiza*, biomass, physiological parameters

## Abstract

An innovative approach to the management of waste in the form of ash obtained during biomass combustion is justified due to its specific properties, including the presence of macro- and microelements. The aim of the current study was to determine the concentration of ash obtained from *Sorghum* combustion regarding its fertilizer value and its effect on the cytological structures, physiological parameters, growth and development of Lemnaceae plants, thereby demonstrating the possibility of using this waste to supplement culture media. The analyses showed that the use of ash in the in vitro cultivation of Lemnaceae aquatic plants had a dose-dependent effect. The addition of 2% ash favorably affected the condition of plant roots, i.e., meristem elongation and an increase in nucleoli sizes as well as improving the chlorophyll content index, gas exchange parameters, chemical oxygen demand (COD) and plant vigor via PSII, which was confirmed by a chlorophyll fluorescence measurement. On the other hand, too high of a concentration, i.e., 10% ash, adversely affected the plant development and parameters studied. Concluding, the use of ash at a low concentration favorably affected the yielding of *Spirodela polyrrhiza*, whose biomass can be used for energy purposes in the production of bioethanol, plant biogas or the phytoremediation of industrial waters and leachate.

## 1. Introduction

Increasing the share of renewable energy sources (RES) is necessary to achieve the European Union (EU) climate and energy goals. The EU is to have 20% RES energy in 2020 and at least 32% by 2030. The energy balance of EU countries assumes that in the coming years, several dozen percent of energy consumed will come from renewable sources, including the biomass of energy plants [1]. Combustion of biomass itself produces a by-product, ash, whose properties are not well studied, especially in the context of agricultural use. The biggest problem is the analysis and development of appropriate technology to manage this waste [2]. The use of ash requires careful assessment and thorough chemical control. On the one hand, ashes are a source of nutrients, macro- and microelements for plants, and they also have deacidifying properties (they can be used as a substitute for calcium fertilizers), while on the other hand, there are some disadvantages associated with the use of this waste, including the presence of harmful substances (heavy metals). There seem to be premises concerning the significant benefits that result from obtaining fertilizer components from the waste products of biomass combustion. It would be unreasonable from an environmental point of view to direct ash into landfills when its valuable components can be used to fertilize plants and improve soil properties. The use of ash resulting from the combustion of organic matter of plant origin is one of the fundamental assumptions of a modern circular economy [3].

Due to the high content of nutrients in ashes, it seems that they can be used both in agriculture as natural fertilizers, and also as a substitute for a liquid-growing medium for Lemnaceae aquatic plants. The development of an economically advantageous technology for the cultivation of aqueous macrophytes in vitro will significantly contribute to their popularization in many industries. The multidirectional nature of Lemnaceae allows their use as natural indicators of the pollution of aquatic ecosystems. Their phytoremediation properties allow the minimization of negative anthropogenic effects. The high protein content of Lemnaceae justifies their use as an animal feed supplement. Their rapid growth, high primary production and the ability to accumulate starch (up to 70% of dry matter) allow for the use of Lemnaceae as a raw material for the efficient and economically profitable production of bioethanol [2,4,5,6,7,8,9].

The root system of multi-root duckweed (*Spirodela polyrrhiza*) consists of 3–15 roots, each up to 15 mm long, growing from a node located in the central underside of the shoot. In the apical parts of the duckweed roots, covered with sheaths, there are meristematic zones with proliferating cells. Each cell in this zone has a nucleus in the central part with a distinct, large single nucleolus. Morphologically, these zones, along with meristematic cells, are similar to those found in the young seedlings of higher seed plants. In plant biology, root tips with meristematic cells are a model research object for, i.a., studies on the impact of various factors on structural and molecular changes in this root zone [10]. In addition, investigations of nucleoli, including changes in their morphology, provide valuable information about physiological status and metabolic activity, including transcriptional activity, not only regarding the nucleoli themselves but also the meristematic cells [11,12]. To date, there are no cytological studies described in the literature on the effect of sorghum ash on the meristematic cells of *S. polyrrhiza* roots. This information is presented here for the first time.

Therefore, the goal of the current cytological research was to assess the effect of sorghum ash present in the plant growth medium on the morphology of *S. polyrrhiza* root meristems and meristematic cell nucleoli. These parameters may indirectly indicate the physiological condition of root meristematic cells of this plant species grown in the presence of micro- and macroelements derived from sorghum ash.

## 2. Materials and Methods

### 2.1. Plant Growth Conditions and Treatments

Preliminary studies based on observations of the condition of plants, taking into account a wider range of ash concentrations, i.e., 1–10%, allowed the selection of two representative concentrations (2% and 10% ash) for further cytological and physiological studies presented in this work.

The exact experiment was carried out over a 14-day period with the use of *Spirodela polyrrhiza* aquatic plants derived from in vitro cultures from the Department of Plant Ecophysiology at the University of Lodz. Daily observation of morphological traits, counting new shoot segments and physicochemical analyses carried out on the last day indicated the varied sensitivity of *S. polyrrhiza* plants growing on three different liquid media, i.e., Z medium, distilled or tap water. These media were additionally supplemented with 2% and 10% concentrations of ash from sorghum. The Z medium was made by hand and was composed of macro- and micronutrients according to [13].

**Figure 1:**
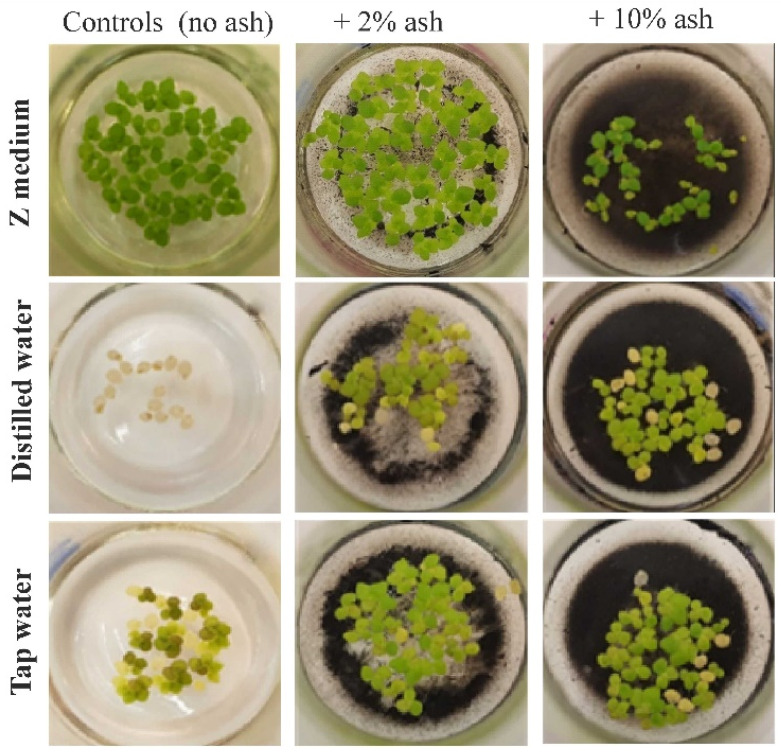
Growth kinetics of *S. polyrrhiza* plants (after 14 days) grown on different medium variants.

The relative growth rate (RGR), i.e., the kinetics of plant growth, was determined by counting all visible leaves (fronds), regardless of their size (Figure 1). Based on the number of leaves on day zero (the experiment’s start day) and day fourteen (the last day), RGR was calculated according to the formula:

(ln x2 − ln x1)/t

where x1 is the number of fronds on day zero of the experiment, x2 is the number of fronds on the last (fourteenth) day of the experiment and t is the number of days to run the experiment.

Macrophytes throughout the 14-day period of the experiment were cultured in a phytotron room at 24 °C, under constant lighting with PHILIPS MASTER TL-D lamps, 2 × 18 W/840. *S. polyrrhiza* plants were grown in 250 mL Erlenmayer flasks with 100 mL respective media for each variant in triplicate. On the last day of the experiment, the following analyses were performed: (1) cytological tests concerning root meristems and (2) physiological ones.

Physiological measurements included: (1) the chlorophyll content index in plant leaves was determined using a Minolta SPAD-502 apparatus, Osaka, Japan; gas exchange parameters, i.e., (2) net photosynthesis (µmolCO_2_ m^−2^s^−1^), (3) transpiration (mmolH_2_O m^−2^s^−1^), (4) stomatal conductivity (mmolH_2_O m^−2^s^−1^) and (5) intercellular CO_2_ concentration (µmolCO_2_/mol air) were determined with a TPS-2 camera (PPSystems, Amesbury, MA, USA); (6) chlorophyll fluorescence was determined using a specialized Handy PEA fluorimeter from Hansatech Instruments Ltd., Norfolk, UK; for this test, the plants were adapted in darkness for 20 min. in order to make all reaction centers in photocircuit II open (oxidized) and ready to receive electrons before measurement [14]. All physiological parameters were measured on live plants with the use of appropriate apparatus equipped with the specific measuring head with a clip which is applied to the leaf. More details concerning the above measurements are described by Romanowska et al. [15]. The weight of the fresh biomass of *S*. *polyrrhiza* plants was presented as an average for the variant and calculated per 1 frond.

The data obtained were subjected to statistical analysis using analysis of variance (ANOVA) with the Statistica 12 program. The means of the chosen parameters were grouped by employing Duncan’s test at =0.05 significance level. All analyzed parameters were repeated five times for each replicate.

### 2.2. Z Medium

The Z medium was made by hand in distilled water with macro- and micronutrients according to [13]. The Z medium composition is as follows: macronutrients: NaNO_3_—mg/L; Ca(NO_3_)_2_ × 4H_2_O—59 mg/L; K_2_HPO_4_—31 mg/L; MgSO_4_ × 7H_2_O—25 mg/L; Na_2_CO_3_—21 mg/L; and Fe-EDTA solution—10 mL/l; and micronutrients: H_3_BO_3_—3700 mg/L; MnSO_4_ × 4H_2_O—2230 mg/L; Na_2_WO_4_ × 2H_2_O—33 mg/L; (NH_4_)_6_Mo_7_O_24_ × 4H_2_O—88 mg/L; KBr—119 mg/L; KJ—83 mg/L; ZnSO_4_ × 7H_2_O—287 mg/L; Cd(NO_3_)_2_ × 4H_2_O—154 mg/L; Co(NO_3_)_2_ × H_2_O—146 mg/L; CuSO_4_ × 5H_2_O—125 mg/L; NiSO_4_(NH_4_)_2_SO_4_ × 6H_2_O—198 mg/L; Cr(NO_3_)_3_ × 7H_2_O—37 mg/L; V_2_O_4_(SO_4_)_3_ × 16H_2_O—35 mg/L; and Al_2_(SO_4_)_3_K_2_SO_4_ × 24H_2_O—474 mg/L.

### 2.3. Composition of Sorghum Ash

The ash was obtained by burning sorghum plants grown on digestate waste originating from the biogas plants in Piaszczyna, Poland. Before being used for testing, the ash was sieved through a sieve with a mesh of 2 × 2 mm. The composition of micro- and macroelements in the ash was marked by the licensed laboratory at the National Research Institute of Horticulture (Skierniewice, Poland). The chemical composition of the ash from the sorghum plants was: N—0.48 mg/kg dry weight; P—14,220 mg/kg dry weight; K—163,000 mg/kg dry weight; Ca—38,430 mg/kg dry weight; Mg—9961 mg/kg dry weight; Fe—2330 mg/kg dry weight; Mn—148 mg/kg dry weight; Cu—29.7 mg/kg dry weight; Zn—1148 mg/kg dry weight; and B—61.9 mg/kg dry weight.

### 2.4. Waters Used

Tap water was derived from Lodz Water Company. The water is characterized by the following selected parameters: pH—7.3–7.7; Ca—69–77 mg/L; Mg—5.6–6.3 mg/L; Na—5.41–6.34 mg/L; K—0.96–1.17 mg/L; CL—8.9–11.0 mg/L; SO_4_—20.0–23.0 mg/L; and F, Cr, Cd, Cu, Ni, Pb, Hg, Fe—below the limit of quantification.

Distilled water (Baxter), ion-free, used for injection and drug dissolution, was purchased from a pharmacy.

### 2.5. Cytological Tests

Cytological studies concerned the measurements of the lengths of root tips containing meristem zones and of the sizes of the root meristematic cell nucleoli. To reveal meristematic cells and nucleoli, the plants were stained according to the Howell and Black method [16] with minor modifications. Whole plants were fixed in 2% glutaraldehyde in a phosphate buffer (pH 7.2) for 10 min; after washing in the same buffer, the plants were re-fixed in a mixture of glacial acetic acid and 96% ethanol (1: 3, by volume) for 5 min. The above procedure was carried out at 4 °C. Then, the material was washed with ethanol at room temperature. After hydration in distilled water, the plants were stained with a mixture consisting of two parts 50% AgNO_3_ (aqueous solution) and one part 2% gelatin in 1% formic acid (aqueous solution) for 5 min at 70 °C in the darkness. The color of the preparations was fixed in 5% sodium thiosulfate for 10 min. The apical parts of the roots were cut from the plants and placed on slides in a drop of water and covered with cover slips. The slides were photographed using a Nikon DS Fil camera and Nikon Eclipse E600 light microscope, Tokyo, Japan. The root tip length and nucleolar area measurements were carried out using the ImageJ program. The lengths of the meristem zones were measured in three random young roots with a total length of 5 mm each in each of the three *S. polyrrhiza* plants for each experimental variant (nine roots for each experimental variant). Areas of 30 random nucleoli from one meristem were analyzed in the above replicates (270 nucleoli for each experimental variant). The results in terms of statistics were analyzed using a one way ANOVA test with Tukey post-hoc comparisons and the GraphPad Prism 7 computer program for statistics; *p* < 0.05 was considered as statistically significant.

## 3. Results

### 3.1. Growth Rate and Fresh Biomass

**Figure 2:**
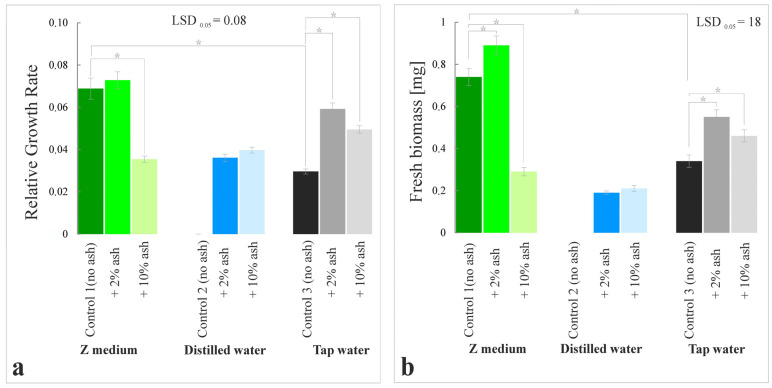
Relative growth rate (RGR) (**a**) and fresh biomass (**b**) in *S. polyrrhiza* plants grown on different variants of sorghum ash-supplemented media. Bars denote means ± SE; asterisks show statistically significant differences between particular series; LSD at alpha level of 0.05.

The growth of macrophytes in the series with the Z medium supplemented with 2% ash was higher compared to Control 1 (Z medium alone) by about 8–9%, while in the series with Z medium and 10% ash, it was lower by 40%. A positive effect was observed in the variant with distilled water supplemented with 2% and 10% ash, where plant growth was higher by about 900% and 1000%, respectively, compared to Control 2 (distilled water alone). Additionally, in the variants with tap water supplemented with 2% and 10% ash, the effects were significant compared to Control 3 (tap water alone), i.e., the growth rate was higher by 220% and by about 170%, respectively (Figure 1 and Figure 2a).

*S. polyrrhiza* grown in the variant with the Z medium with 2% ash was characterized by the highest biomass among all experimental series. It was higher by 27% compared to the control (Control 1), whereas the plants grown in the 10% ash variant were reduced by 59% compared to Control 1. A high response of *S. polyrrhiza* plants was observed in the series with distilled water supplemented with 2% and 10% ash, where the fresh mass was higher by 600% and 700%, respectively, compared to Control 2. Macrophytes grown on tap water with the addition of 2% and 10% ash were characterized by a higher biomass by 60% and 35%, respectively, compared to Control 3 (Figure 2b).

### 3.2. Lengths of Root Tips

**Figure 3:**
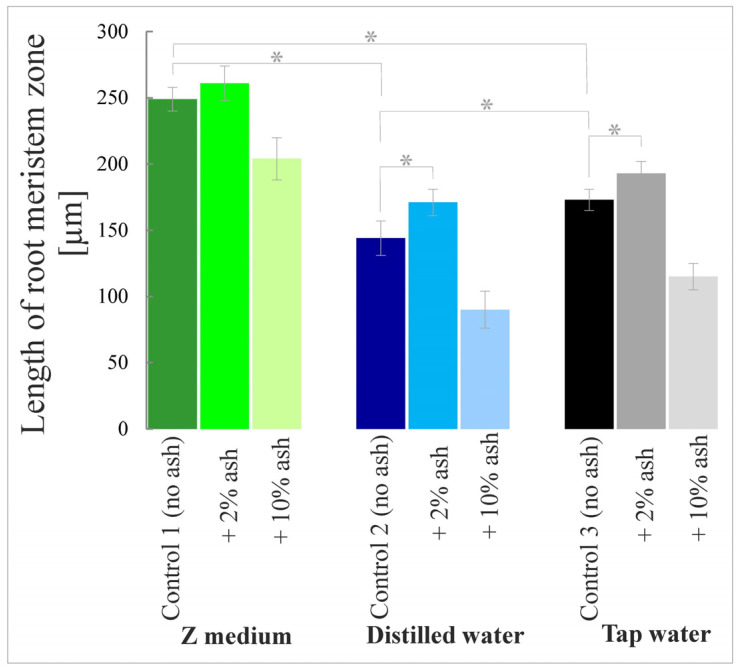
Lengths of the meristematic zones of *S. polyrrhiza* roots grown in respective media; asterisks show statistically significant differences between particular root lengths at *p* < 0.05; bars represent standard deviations (average lengths ± SD).

**Figure 4:**
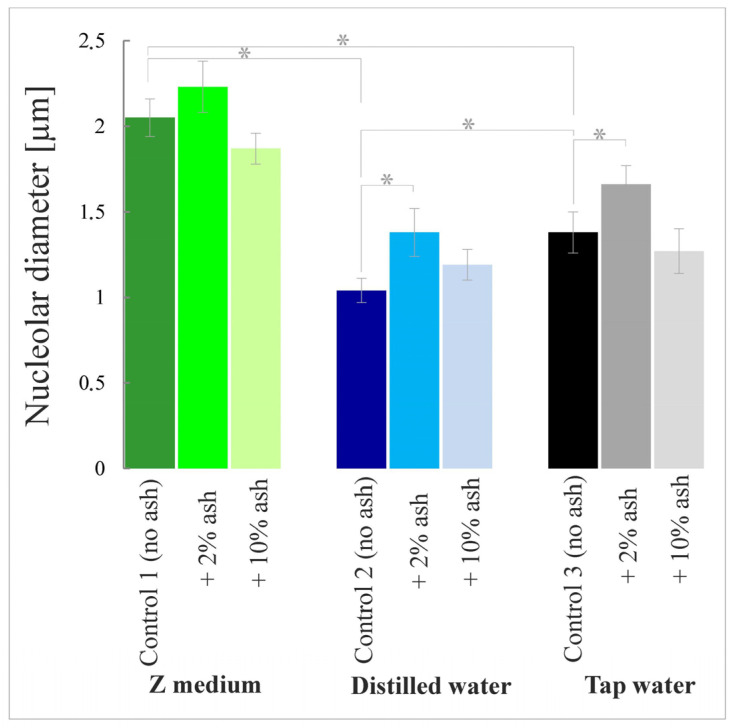
Sizes (diameters) of nucleoli in meristematic cells of *S. polyrrhiza* root tips grown in respective media; asterisks show statistically significant differences between particular root lengths at *p* < 0.05; bars represent standard deviations (average sizes ± SD).

Out of all controls, i.e., the variants without ash, the longest meristematic zones in the root tips were in plants grown in the Z medium (Control 1) (Figure 3 and Figure 4). The root meristematic parts of plants grown in distilled water (Control 2) or tap water (Control 3) were shorter compared to Control 1 by 42.2% and 30.5%, respectively.

The addition of 2% ash resulted in the lengthening of the meristematic parts by 4.8%, 18.8% and 11.6%, compared to the respective control, i.e., to Control 1, 2 or 3. These results may suggest that supplementation with 2% ash improves the meristem condition in relation to the environment without ash; however, there was no statistically significant difference between Control 1 (without ash) and Control 1 + 2% ash. In contrast, 10% ash solutions significantly shortened the meristematic parts by 18.1%, 37.5% and 33.5%, compared to the respective control, i.e., to Control 1, 2 or 3. Such a result proves that a too high ash concentration is unfavorable for the development of the meristematic zone of *S. polyrrhiza* plant roots. These tests also showed that distilled water impaired the development of the meristematic zone of the roots of duckweed in relation to the plants grown in the Z medium or in tap water (Figure 3 and Figure 4).

### 3.3. The Sizes of Nucleoli

Among the controls without ash, the largest nucleoli, i.e., the largest in diameter, were observed in meristematic cells of the plant roots grown in the Z medium (Control 1). The smallest nucleolar diameters were in the plant cells grown in distilled water alone (Control 2). Their average diameter was smaller than those in Control 1 by almost half (49.3%). The average nucleolar diameter in plants grown in tap water alone was smaller by 32.7% in comparison to Control 1.

**Figure 5:**
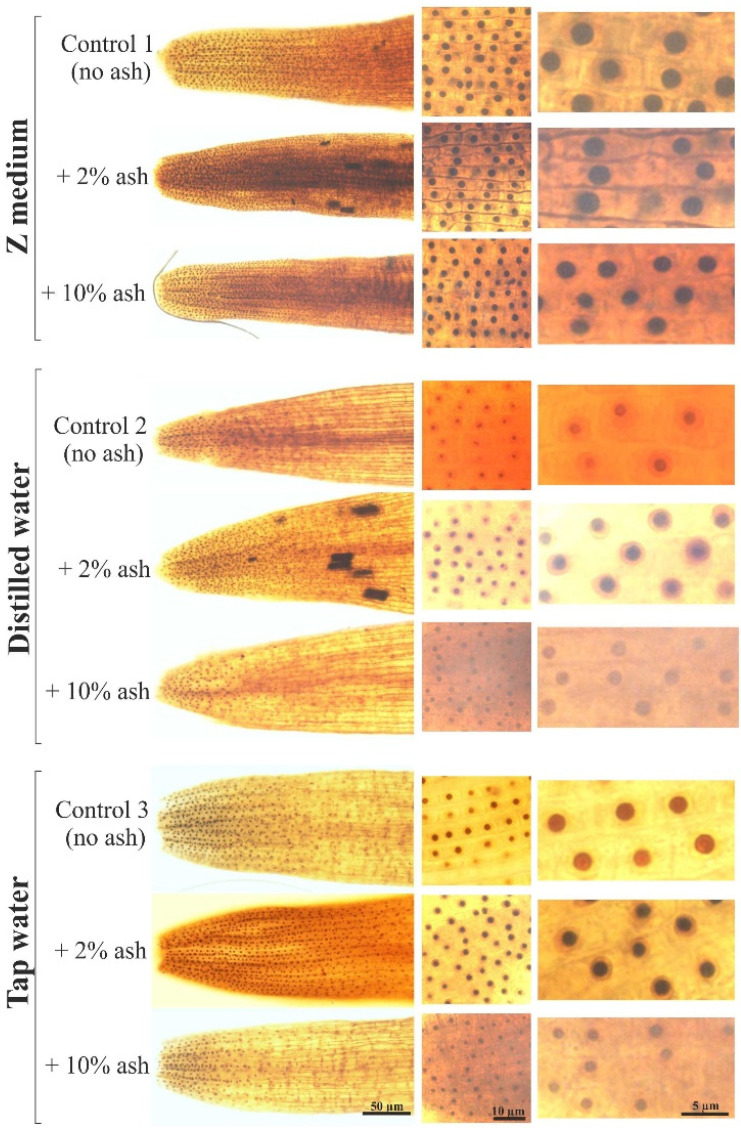
Root apexes (left panel) and fragments of meristematic zones with cells (middle panel) with clear nucleoli (right panel) in *S. polyrrhiza* plants grown in studied media.

The addition of 2% ash caused an increase in nucleolar diameters by 8.8%, 32.7% and 20.3%, in comparison to the respective control, i.e., to Control 1, 2 or 3 (Figure 4 and Figure 5). The higher ash concentration, i.e., 10% ash, decreased nucleolar sizes by 8.8% and 8.0% in comparison to the respective control, i.e., to Control 1 or 3, whereas it increased the nucleolar diameter by 14.4% in relation to Control 2. In the two former cases, the differences were statistically insignificant.

These results may indicate that the Z medium very advantageously influences nucleolar condition in contrast, especially, to distilled water alone, which adversely affects the functioning of nucleoli in *S. polyrrhiza* root cells. The addition of ash in lower concentrations seems to improve the physiological state of the nucleoli.

### 3.4. Index of Chlorophyll Content

**Figure 6:**
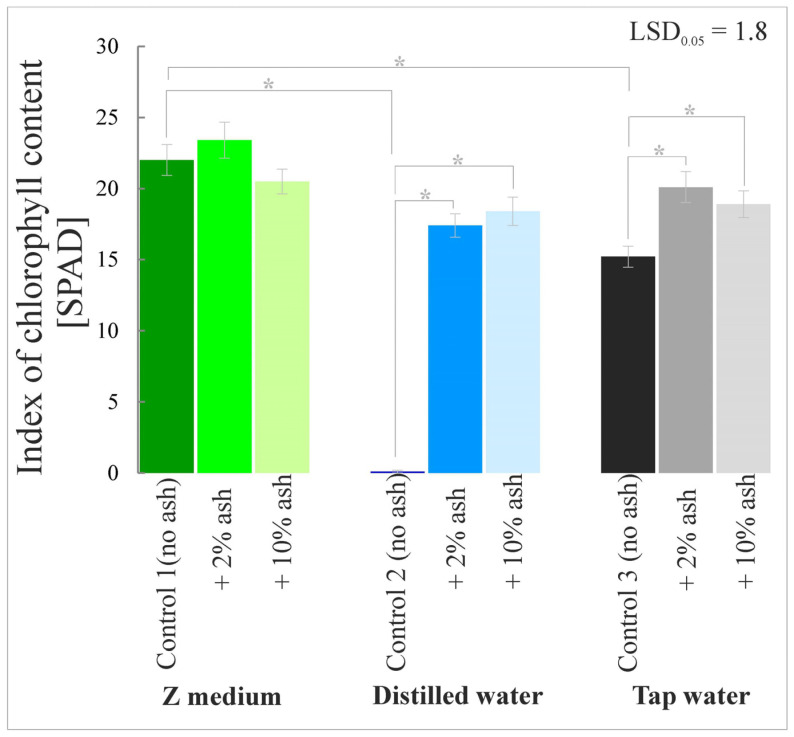
Index of chlorophyll content in *S. polyrrhiza* plants grown on different variants of ash-supplemented media. Bars denote means ± SE; asterisks show statistically significant differences between particular series; LSD at alpha level of 0.05.

The chlorophyll (a + b) content index in macrophyte leaves in the variant with the Z medium with 2% ash was higher by more than 6% compared to Control 1. The highest increases in the index values were recorded for the variants with distilled water with 2% and 10% ash; on average they were 17.4 and 18.4 band, respectively. *S. polyrrhiza* grown in tap water supplemented with ash in comparison to Control 3 was characterized by a higher content of chlorophyll in the leaves by 32% and 24% for 2% and 10% ash, respectively (Figure 6).

### 3.5. Chemical Oxygen Demand (COD)

**Figure 7:**
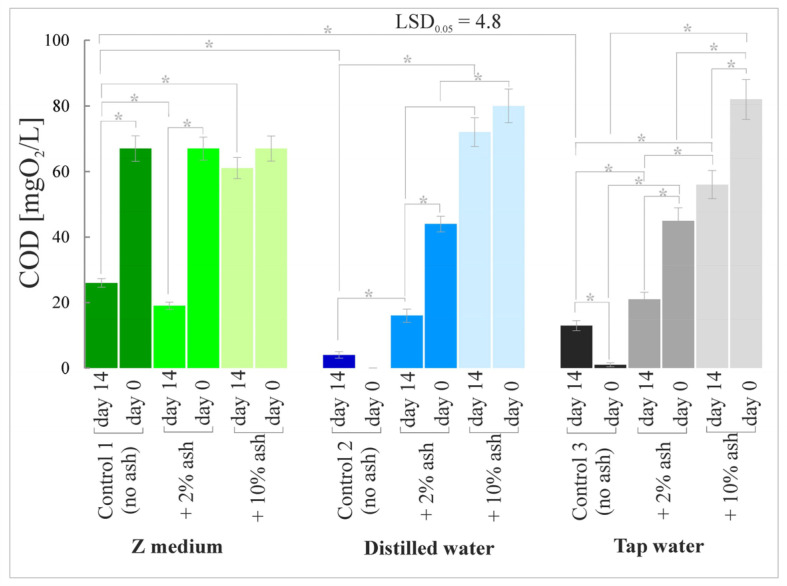
Chemical oxygen demand (COD) in *S. polyrrhiza* plants grown on different variants of ash-supplemented media. Bars denote means ± SE; asterisks show statistically significant differences between particular series; LSD at alpha level of 0.05.

The amount of oxygen taken from the oxidant in order to oxidate organic and inorganic compounds present in water varied for individual experimental variants and its highest level was observed in the variants with 10% ash (Figure 7).

### 3.6. Chlorophyll Fluorescence

**Figure 8:**
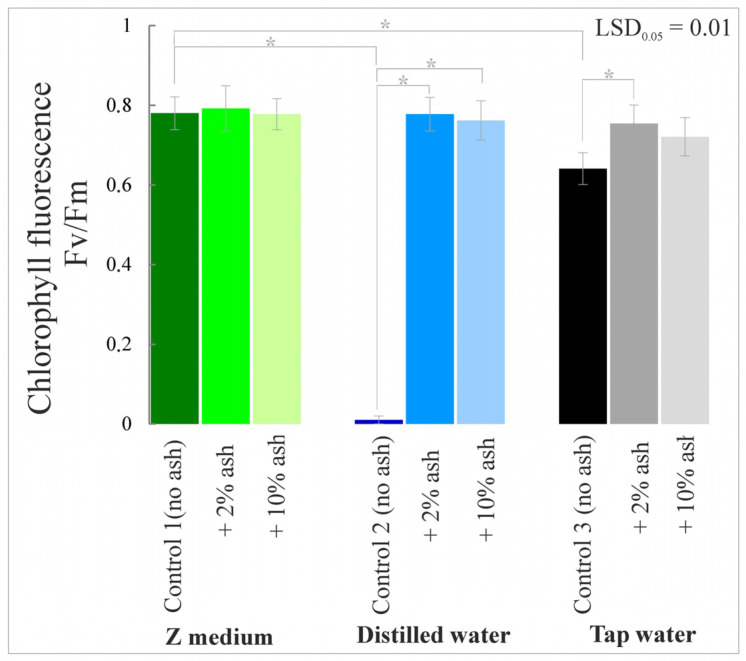
Chlorophyll fluorescence in *S. polyrrhiza* plants grown on different variants of ash-supplemented media. Bars denote means ± SE; asterisks show statistically significant differences between particular series; LSD at alpha level of 0.05.

The highest chlorophyll fluorescence was characteristic of the plants grown in the Z medium supplemented with 2% and 10% ash (Figure 8). Low levels of Fv/Fm in the variants with tap water and distilled water indicate a lower efficiency of the PSII photosystem in the darkness and reduced demand of macrophytes for products constituting the so-called assimilation strength under stress conditions. Supplementing the medium with macro- and microelements present in the ashes caused better plant development, which translated into a higher level of chlorophyll fluorescence. The variant with tap water and 2% or 10% ash was characterized by a relatively high value of the Fv/Fm parameter (17% and 15% higher compared to Control 3), which proves the high potential efficiency of photosystem II. Previous analyses using this parameter indicate that chlorophyll fluorescence may be used as an indicator of plant damage caused by various environmental factors, especially stress arising from nutrient deficiency.

### 3.7. Gas Exchange Parameters

**Figure 9:**
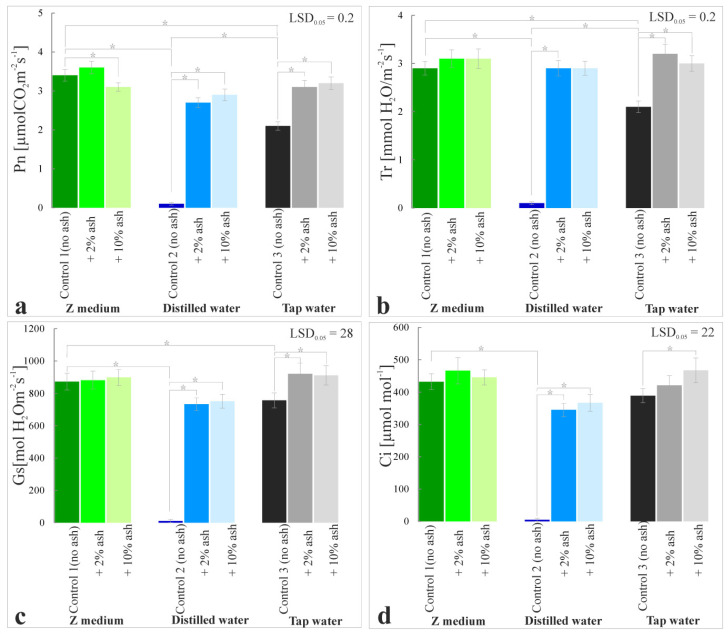
Net photosynthesis, Pn (**a**), transpiration, Tr (**b**), stomatal conductance, Gs (**c**) and intercellular concentration of CO2, Ci (**d**) in the leaves of *S. polyrrhiza* plants grown on different variants of ash-supplemented media. Bars denote means ± SE; asterisks show statistically significant differences between particular series; LSD at alpha level of 0.05.

The analysis of gas exchange parameters demonstrated the beneficial effects of ash supplementation on the development of S. polyrrhiza. In the series grown in the Z medium, the most favorable net effect and value of photosynthesis (Figure 9a) were observed for the variant with 2% ash, and it was higher by 6% compared to Control 1 and slightly lower, i.e., by 9%, for the 10% ash variant. The value of net photosynthesis in the plants grown in distilled water with the addition of 2% or 10% ash was significantly increased relative to Control 2 and it was 2.7 and 2.9 µmolCO_2_ m^−^^2^s^−^^1^ on average, respectively. An upward trend was also observed in the series with tap water supplemented with 2% and 10% ash and in these variants, the values were 47% and 52% higher, respectively, in comparison to Control 3.

The intensity of the transpiration process observed in the variants cultivated in the Z medium with 2% and 10% ash remained at a similar level as Control 1 and ranged from 2.9 to 3.1 mmolH_2_O m^−^^2^s^−^^1^ (Figure 9b). A significant increase in transpiration was observed in the distilled water series and was 2.9 mmolH_2_O m^−^^2^s^−^^1^ for both 2% and 10% ash, while for the variants with tap water with 2% and 10% ash, these values were higher by 50% and 42%, respectively, compared to Control 3.

The stomatal conductivity is an important indicator of the plant water status and provides important information about the development of plants and their adaptation to changing environmental conditions. *S. polyrrhiza* grown in the Z medium with or without ash was characterized by similar stomatal conductivity (873–899 mmolH_2_O m^−^^2^s^−^^1^). The stomatal conductivity of plants grown on ash-supplemented distilled water was 734–752 mmolH_2_O m^−^^2^s^−^^1^, while on distilled water alone it was only 10 mmolH_2_O m^−^^2^s^−^^1^. An increase in the intensity of this process was observed in the group of macrophytes grown in tap water with 2% and 10% ash; this parameter was higher by 21% and 19%, respectively, compared to Control 3 (Figure 9c).

The gas exchange parameter was shown as the intercellular concentration of CO_2_ (Figure 9d). The increase in nutrient content in the medium, and consequently better plant development, translated into higher levels of intercellular CO_2_ concentration. In the series of plants growing on the Z medium with the addition of 2% and 10% ash, this parameter was higher by 8% and 7%, respectively, compared to Control 1. A clear increase in intercellular CO_2_ concentrations was observed in the variant with distilled water with ash, and it was in the range of 345–367 µmolCO_2_/mol air, but in distilled water without ash (Control 2) it was only 5 µmolCO_2_/mol air. Macrophytes grown in tap water with the addition of 2% and 10% ash had better intercellular CO_2_ concentrations and it was higher by 8% and 12%, respectively, compared to Control 3.

## 4. Discussion

Positive and negative environmental conditions directly and indirectly affect plant growth, differentiation and development through changes in cellular processes. Terrestrial and aquatic plants are exposed throughout life to various environmental factors that disrupt homeostasis and cause cellular stress. The detection of stressful signals and an appropriate response leads to a modification of physiological processes, which determine the adaptation to new conditions and, consequently, the survival of an organism [2,6,7,8,9].

In addition, signals, including those associated with environmental reactions, transduced between different plant organs, can change the processes associated with cell growth and proliferation in meristems. Signal perception and response are defined as meristem competence, which is responsible for cell growth and division, and ultimately for organ growth, including roots [17,18]. The growth of proliferating cells is driven by continuously synthesized proteins in large quantities, which is provided by an abundance of ribosomes. Therefore, the proliferative activity of meristematic cells, and thereby growth of organs, is directly correlated with the rate of ribosome production in the nucleolus [19,20].

In the current research, the lengths of root tips containing meristems were measured, and the sizes of the nucleoli of root meristematic cells of duckweed grown on the Z medium, distilled or tap water alone and with the addition of 2% and 10% ash, were determined. The main control was the plants grown in the Z medium, rich in all necessary nutrients. Analysis of the individual parameters allowed for the indirect assessment of the metabolic and transcriptional activity of cells. The volume occupied by the meristematic zone, which depends on the number of meristematic cells and intensity of their divisions, affects the rate of growth and development of the entire root length [21,22]. A larger meristematic zone with intensively dividing cells may indicate favorable conditions for plant growth and development [23,24].

It has been proven that the morphology and size of nucleoli are linked to nucleolar activity. Various negative cellular factors, e.g., nutritional status or environmental conditions, may affect this activity. The sensitivity of nucleoli to these insults is manifested by disturbances in ribosome biogenesis, which is often accompanied by morphological disorders of the nucleolus [25,26] and this phenomenon is called nucleolar/ribosomal stress [27]. Therefore, it is believed that the nucleolus is a key player in the detection and response to cellular stress in various systems, including animals and humans [28,29,30,31,32].

The activity, size and shape of the nucleolus are closely correlated with the growth and proliferation of the eukaryotic cells and reflect the demand for proteins. Thus, the nucleolar structure, including its organization and size, which are easy to detect even at the level of a light microscope, has been recognized as an effective and reliable indicator of cellular stress and growth conditions [27,33] and as a useful parameter determining the functional activity of nucleoli, mainly in relation to ribosome biosynthesis, and in consequence is considered as a parameter determining the rate of cell growth and proliferation [19]. Large nucleoli are usually present in intensively dividing, metabolizing and highly protein-biosynthesizing cells, which is associated with increased rDNA transcription and, consequently, with a large number of ribosomes. Such a scenario occurs, among others, in the transformed cells of many types of cancer [34]. The opposite situation (occurrence of small nucleoli) is characteristic of cells with low metabolic activity [35]. The impact of stressors, such as nitrogen or glucose deficiency, heat or oxidative stress or UV radiation causes serious structural reorganization of the nucleoli into smaller, compact or disintegrating ones, which is accompanied by inhibition of rRNA biosynthesis and nucleolar protein relocation [32]. Although the efficiency of ribosome biosynthesis and cell proliferative activity generally coexist and correlate with the environmental parameters of plant growth and development, special conditions can separate cell proliferation and ribosome biosynthesis [19].

There is growing evidence that nucleolar activity is directly or indirectly related to the condition of plants, including exposure to biotic and abiotic stresses [36]. It was shown that disorders of any stage of ribosome biogenesis caused an impairment to some aspect of plant growth and development. It is believed that the reduced growth and development of plants deficient in nucleolar proteins or ribosome-related factors causes a reduction in cell divisions and overall protein failure due to ribosome deficiency in meristematic tissues [37,38,39]. Recently, it has been suggested that stress-induced phenotypic changes in plant organs result from the activation of plant-specific signaling pathways in which the cellular stress response was involved [40,41].

The current research in which the root meristem zones and nucleoli of meristematic cells were analyzed showed that the Z medium is the most favorable medium for the growth and development of *S. polyrrhiza* plants among the media without ash. Distilled water alone, without trace elements, adversely affected the development of the macrophyte root zone. In contrast, tap water, which contains a certain amount of microelements, provided better growth conditions than distilled water alone. Supplementation of the Z medium, distilled or tap water with a low dose of ash (2%) significantly improved the analyzed parameters, i.e., root meristem lengths and nucleolar sizes, which translates into better growth and development of the plants. Analyses of gas exchange parameters, chlorophyll content index, chlorophyll fluorescence and biomass amount coincided with positive effects in the root meristem zone.

Paliya et al. [42] obtained similar results, using ash as a fertilizer for lentil seeds. Leachate ash was shown to have a positive effect on the growth, development and germination of lentil seeds and on plant growth and vigor. They also showed that ash from sewage sludge could be used as a biological fertilizer for *Rhizobium*—living in symbiosis with papilionaceous plants that form growths on their roots, the so-called root warts that have a significant effect on root length, root and shoot dry mass, number and dry mass of root nodules. In addition, it was demonstrated that the increase in germination of seeds treated with biofertilizer from sludge ashes could be caused by the presence of increased amounts of macro- and microelements, including Mn, Fe, Cu and Zn, derived from sediments.

In many cases, fly ash is considered as an environmental threat because it contains organic pollutants as well as toxic metals such as Se, As, B, V, Al, Pb, Hg, Cr, and even Ra, U and Th. These ashes can also contain toxic substances and most of the oxides and trace elements that contribute to an alkaline pH, while the trace elements provide nutrients for plant growth. That is why many authors suggest that ash can be used at low concentrations in the agricultural sector, such as phytoremediation, bioremediation, wasteland reclamation and forestry. In addition, it can be used as a means to improve soil quality, by improving the physicochemical and biological properties of polluted land [43]. Fly ash from coal, due to its physical and chemical properties, influences biological processes, and thus affects plant growth and development [44,45,46]. Many studies showed that the addition of ashes to the soil generally increased plant growth including turf grass [47], wheat [48], rice [49] or mustard [50]. The best beneficial effects of ash on plants and an increase in productivity are usually demonstrated when the ashes are weathered, which is related to pH, salinity and phytotoxicity.

Most of the research, however, is based on coal ashes, while research on fly ash from plant biomass has been rarely reported in the literature and should be particularly taken into account. In the work of Niu et al. [51], it was suggested that ash could be used as a means of improving soil quality or as a fertilizer. It should also be noted that the fate of ash-related nutrients (micro- and macroelements), their release rate and possible availability for uptake by plants, as well as the presence of alkali and alkaline soil metals are virtually unknown. Therefore, research and tests should be focused on acquiring knowledge in this area.

## 5. Conclusions

Supplementation of the cultivation medium (soil or water) with the ash from sorghum plant burning can favorably affect the growth and development of plants, and thus may result in greater biomass and higher yields. Macrophytes were characterized by better growth and higher physicochemical parameters when grown on Z medium, tap or distilled water supplemented with the ash. Cheap and widely available tap water with the addition of sorghum ash rich in macro- and microelements can be successfully used as a medium for growing Lemnaceae plants instead of using expensive Z medium or other chemical nutrients. However, it must be borne in mind that Z medium, unlike ash, is well balanced in terms of the content of necessary nutrients, while ash is much richer in some components (e.g., Mg or K) and poorer in others (e.g., N). Therefore, ash should be used prudently in small doses, e.g., 2% ash, as this work showed. Importantly, the chemical composition of ash obtained from the combustion of plant biomass can vary significantly between individual supplies of this waste, and therefore each batch of ash should be subjected to chemical control and toxicity tests.

Concluding, the current studies indicate the possibility of using ash in plant cultivation; thereby, they propose the management of a problematic by-product of the burning of energy-rich renewable plant biomass, which is part of the latest assumptions of the circular economy of the 21st century.

## Data Availability

Not applicable.
